# SLC38A8 mutations result in arrested retinal development with loss of cone photoreceptor specialization

**DOI:** 10.1093/hmg/ddaa166

**Published:** 2020-08-03

**Authors:** Helen J Kuht, Jinu Han, Gail D E Maconachie, Sung Eun Park, Seung-Tae Lee, Rebecca McLean, Viral Sheth, Michael Hisaund, Basu Dawar, Nicolas Sylvius, Usman Mahmood, Frank A Proudlock, Irene Gottlob, Hyun Taek Lim, Mervyn G Thomas

**Affiliations:** The University of Leicester Ulverscroft Eye Unit, Department of Neuroscience, Psychology and Behaviour, University of Leicester – RKCSB, PO Box 65, Leicester LE2 7LX, UK; Institute of Vision Research, Department of Ophthalmology, Gangnam Severance Hospital, Yonsei University College of Medicine, Seoul 06273, Korea; The University of Leicester Ulverscroft Eye Unit, Department of Neuroscience, Psychology and Behaviour, University of Leicester – RKCSB, PO Box 65, Leicester LE2 7LX, UK; Academic Unit of Ophthalmology and Orthoptics, University of Sheffield, Sheffield S10 2RX, UK; Institute of Vision Research, Department of Ophthalmology, Gangnam Severance Hospital, Yonsei University College of Medicine, Seoul 06273, Korea; Department of Laboratory Medicine, Severance Hospital, Yonsei University College of Medicine, Seoul 06273, Korea; The University of Leicester Ulverscroft Eye Unit, Department of Neuroscience, Psychology and Behaviour, University of Leicester – RKCSB, PO Box 65, Leicester LE2 7LX, UK; The University of Leicester Ulverscroft Eye Unit, Department of Neuroscience, Psychology and Behaviour, University of Leicester – RKCSB, PO Box 65, Leicester LE2 7LX, UK; The University of Leicester Ulverscroft Eye Unit, Department of Neuroscience, Psychology and Behaviour, University of Leicester – RKCSB, PO Box 65, Leicester LE2 7LX, UK; The University of Leicester Ulverscroft Eye Unit, Department of Neuroscience, Psychology and Behaviour, University of Leicester – RKCSB, PO Box 65, Leicester LE2 7LX, UK; Department of Genetics and Genome Biology, University of Leicester, Leicester LE1 7RH, UK; Department of Ophthalmology, Hull and East Yorkshire Hospitals NHS Trust, Hull HU3 2JZ, UK; The University of Leicester Ulverscroft Eye Unit, Department of Neuroscience, Psychology and Behaviour, University of Leicester – RKCSB, PO Box 65, Leicester LE2 7LX, UK; The University of Leicester Ulverscroft Eye Unit, Department of Neuroscience, Psychology and Behaviour, University of Leicester – RKCSB, PO Box 65, Leicester LE2 7LX, UK; Department of Ophthalmology, Asan Medical Center, University of Ulsan College of Medicine, Seoul 05505, Korea; The University of Leicester Ulverscroft Eye Unit, Department of Neuroscience, Psychology and Behaviour, University of Leicester – RKCSB, PO Box 65, Leicester LE2 7LX, UK

## Abstract

Foveal hypoplasia, optic nerve decussation defects and anterior segment dysgenesis is an autosomal recessive disorder arising from *SLC38A8* mutations. SLC38A8 is a putative glutamine transporter with strong expression within the photoreceptor layer in the retina. Previous studies have been limited due to lack of quantitative data on retinal development and nystagmus characteristics. In this multi-centre study, a custom-targeted next generation sequencing (NGS) gene panel was used to identify *SLC38A8* mutations from a cohort of 511 nystagmus patients. We report 16 novel *SLC38A8* mutations. The sixth transmembrane domain is most frequently disrupted by missense *SLC38A8* mutations. Ninety percent of our cases were initially misdiagnosed as *PAX6*-related phenotype or ocular albinism prior to NGS. We characterized the retinal development *in vivo* in patients with *SLC38A8* mutations using high-resolution optical coherence tomography. All patients had severe grades of arrested retinal development with lack of a foveal pit and no cone photoreceptor outer segment lengthening. Loss of foveal specialization features such as outer segment lengthening implies reduced foveal cone density, which contributes to reduced visual acuity. Unlike other disorders (such as albinism or *PAX6* mutations) which exhibit a spectrum of foveal hypoplasia, *SLC38A8* mutations have arrest of retinal development at an earlier stage resulting in a more under-developed retina and severe phenotype.

## Introduction

Phenotypical characteristics of foveal hypoplasia (FH) and abnormalities of optic nerve decussation have previously been described in association with albinism and albinism syndromes due to disruption of the melanin biosynthesis pathway (1). Al-Araimi et al. described a new recessively inherited disorder called FH, optic nerve decussation defects and anterior segment dysgenesis (FHONDA), which had significant overlap of phenotypical characteristics with albinism. However, in FHONDA, there were no features of cutaneous or ocular hypopigmentation (2). Poulter et al. identified that mutations of *SLC38A8*, a putative glutamine transporter gene found on chromosomes 16q23.3–24.1, were causative of FHONDA (3). This is thought to be part of a melanin-independent pathway affecting foveal development.

**Figure 1:**
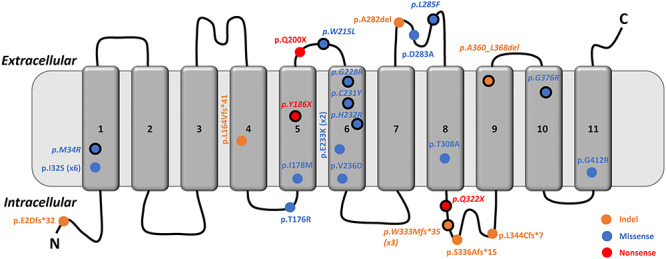
(**A**) Schematic representation of the SLC38A8 protein with 11 transmembrane domains and location of amino acid changes in relation to the protein domains. Mutations in italics and with a solid black outline are ones reported in this study. Transmembrane domain 6 has recurrent missense mutations. c.995dupG: p.(W333Mfs*35) was a recurrent frameshift mutation reported in this study that was observed in three separate Korean families. Similarly, p.I32S is a recurrent mutation (reported in six separate families) observed in both Indian and Karaite Jewish ethnicities.

To date, 17 *SLC38A8* mutations have been described (3,4,5,6,7). The mutational spectrum is varied (Fig. 1), and includes missense, nonsense and frameshift mutations. Splice mutations have not been described. Infantile nystagmus (IN) and FH are the most consistent phenotypes observed in all previously described families. Optic nerve decussation defects are also likely to be a consistent phenotype, however, not reported in all cases due to lack of visual evoked potentials (VEP) in some cases (4,5). It is hypothesized that more deleterious mutations may be associated with anterior segment dysgenesis (ASD) (5). Recently, two patients with FHONDA were reported to have iris transillumination defects (TID). Both with compound heterozygous mutation of *SLC38A8*; with an amino acid substitution at Thr308 (6). This further blurs the phenotypical overlap between albinism and FHONDA. The detailed phenotypical characteristics including nystagmus features and degree of arrested retinal development in *SLC38A8* mutations have not been systematically analyzed in relation to genotypes.

We studied 11 subjects from nine families with *SLC38A8* mutations, which represents the largest number of families with *SLC38A8* mutations reported and describe for the first time the detailed eye movement characteristics, foveal developmental abnormalities and expand the genotypic spectrum of *SLC38A8* mutations.

## Results

### Genetics

We identified a total of 17 *SLC38A8* mutations in 11 affected subjects from nine families (Table 1). The American College of Medical Genetics (ACMG) classification of the variants and segregation analyses are provided in Table 1. Sixteen of these mutations were novel and one (c.101 T > G; p.(Met34Arg)) previously reported (2). The location of each of the mutations is shown in the schematic in Figure 1. Of the nine families, two had homozygous mutations (F7: c.101 T > G; p.(Met34Arg) and F8: c.632 + 2 T > G). The remaining families (F1–F6), Korean in ethnicity, had compound heterozygous mutations. One Caucasian British family (F9) also had a compound heterozygous mutation.

**Table 1:** SLC38A8 mutations identified

Pedigree ID	Patient ID	Gene	Mutation (s)	Zygosity	gnomAD (MAF)	CADD	FATHMM	ACMG	Segregation	Previous literature
F1	F1:II-1 and F1:II-2	*SLC38A8*	c.692G > A:p.(Cys231Tyr)	Compound heterozygous	1/223 834	27.2	0.96	LP	Paternal	Novel
		*SLC38A8*	c.964C > T:p.(Gln322^a^)		2/248 656	37.0	0.97	P	Maternal	Novel
F2	F2:II-1	*SLC38A8*	c.558C > A:p.(Tyr186^a^)	Compound heterozygous	None	58.0	0.93	P	Maternal	Novel
		*SLC38A8*	c.1078_1104del:p.(Ala360_Leu368del)		None	16.3	0.99	LP	Paternal	Novel
F3	F3:II-1	*SLC38A8*	c.995dupG:p.(Trp333Metfs^*^35)	Compound heterozygous	1/249 760	22.9	0.72	P	Paternal	Novel
		*SLC38A8*	c.1214 + 5G > C		None	14.0	0.99	US	Maternal	Novel
F4	F4:II-1	*SLC38A8*	c.855G > C:p.(Leu285Phe)	Compound heterozygous	None	35.0	0.67	LP	Maternal	Novel
		*SLC38A8*	c.995dupG:p.(Trp333Metfs^*^35)		1/249 760	16.3	0.72	P	Paternal	Novel
F5^a^	F5:II-1 and F5:II-2	*SLC38A8*	c.644G > T:p.(Trp215Leu)^c^	Compound heterozygous	10/251 306	33.0	0.97	US	NA	Novel
		*SLC38A8*	c.682G > A:p.(Gly228Arg)^c^		7/282 722	32.0	0.99	US	NA	Novel
		*SLC38A8*	c.695A > G:p.(His232Arg)		1/234 770	23.7	0.95	US	Maternal	Novel
F6	F6:II-2	*SLC38A8*	c.954-1G > C	Compound heterozygous	None	23.5	0.98	P	Detected in trans^d^	Novel^e^
		*SLC38A8*	c.995dupG:p.(Trp333Metfs^*^35)		1/249 760	16.3	0.72	P	Detected in trans^b^	Novel
F7	F7:II-3	*SLC38A8*	c.101 T > G:p.Met34Arg	Homozygous	None	17.2	0.99	LP	Maternal and Paternal^d^	Reported^e^
F8^b^	F8:II-1	*SLC38A8*	c.632 + 2 T > G	Homozygous	None	22.9	0.99	P	Maternal and Paternal	Novel
F9	F9:II-1	*SLC38A8*	Exon 1 deletion	Compound heterozygous	None	-	-	P	Maternal	Novel
		*SLC38A8*	c.1126G > A:p.(Gly376Arg)		2/2 282 530	27.1	0.96	LP	Paternal	Novel

^a^Additional variant in F5: *FRMD7* (NM_194277.2)*:* c.875 T > C:p.(Leu292Pro) (heterozygous and hemizygous in F5:II-1 and F5:II-2, respectively).

^b^Additional variant in F8: *TYR* (NM_000372): c.1205G > A:p.(Arg402Gln) (homozygous).

^c^These two variants were present in cis configuration, confirmed by IGV.

^d^These two variants were present in trans configuration, confirmed by IGV.

^e^Poulter, J.A., Al-Araimi, M., Conte, I. et al. Recessive mutations in *SLC38A8* cause foveal hypoplasia and optic nerve misrouting without albinism. Am J Hum Genet. 2013, 1143–1150.

Abbreviations: ACMG = American College of Medical Genetics; CADD = combined annotation dependent depletion; FATHMM = functional analysis through Hidden Markov Models; LP = likely pathogenic; NA = not available; P = pathogenic and US = uncertain significance.

**Figure 2:**
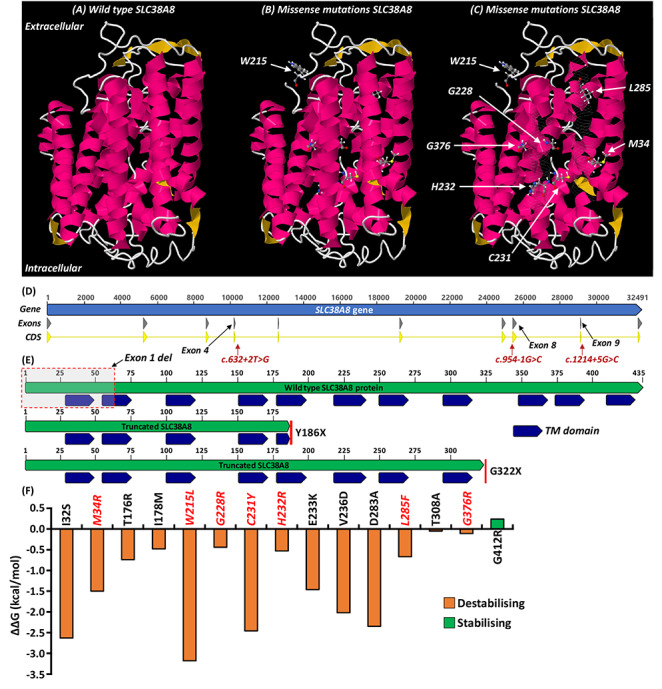
(**A**) Predicted wild type SLC38A8 protein 3D structure. (**B** and **C**) Location of the missense mutations resulting in amino acid substitutions at residue positions 34, 215, 228, 231, 232, 285 and 376. The residues 59–74 and 252–274 have been made see-through (C) to allow visualization of inner structure including transmembrane domain 6. Residue 215 is located within a coil region in the extracellular area between transmembrane domains 5 and 6. (**D**) SLC38A8 gene structure and location of novel splice mutations. (**E**) Schematic depicts location of nonsense mutations resulting in truncated protein. (**F**) Amino acid substitutions and predicted stability change (ΔΔ*G*). The Gibbs free energy gap difference (ΔΔ*G* = Δ*G_m_* – Δ*G_w_*) between the mutant (Δ*G_m_*) and wild type (Δ*G_w_*) protein. Negative changes in ΔΔ*G* were predicted to be destabilizing. Variants reported in this study are shown in red and italics. TM domain = transmembrane domain.

The six missense mutations resulting in amino acid substitutions at positions 215, 228, 231, 232, 285 and 376 not only involve highly conserved residues that are invariant in *Rattus norvegicus*, *Mus musculus*, *Gallus gallus*, *Xenopus tropicalis* and *Danio rerio* (Supplementary Material, Fig. S1) but are also located within invariant blocks of highly conserved residues. Thus suggesting that mutations at these locations are critical to the normal function of the protein. The residues at position 228, 231 and 232 are within the sixth transmembrane domain, which appears to be a commonly affected domain (previous mutations in this domain: Glu233Lys (two separate reports) (3,6) and Val236Asp) (3). Structural modelling shows that all amino acid substitutions, except Trp215Leu, are within α-helices deep within the protein structure. The Trp215 residue is located within a coil region in the extracellular area between transmembrane domains 5 and 6 (Fig. 2). All amino acid substitutions are predicted to be destabilizing (Fig. 2). The six missense mutations all have a CADD score above 20.0 indicating they are predicted to be in the 1% most deleterious substitutions that can be applied to the human genome.

In our cohort, we identified three splice mutations. The c.632 + 2 T > G and c.954-1G > C alters the splice donor (at exon4–intron4 junction) and acceptor (at intron7–exon8 junction) sites, respectively (Fig. 2). Both alter the strongly conserved canonical donor (+1 and + 2 nucleotides) or acceptor (−1 and − 2 nucleotides) sites and are predicted to be pathological by classical exon skipping and nonsense mediated decay (NMD). The c.1214 + 5G > C mutation is predicted (Supplementary Material, Fig. S2) to alter the wild type splice donor site (at exon9–intron9 junction) (Fig. 2), and thus also pathological by exon skipping and NMD.

The nonsense mutations Tyr186* and Gln322* predict truncating proteins containing 43% and 74% of the protein, respectively (Fig. 2). We identified a recurrent frameshift mutation (c.995dupG: p.(W333Mfs*35)) in three Korean families (F3, F4, F6) (Table 1). We identified a deletion (exon 1) (in F9). The frameshift and nonsense mutations resulting in a premature termination codon and truncated protein are predicted be pathological by NMD. Similarly, exon 1 deletion is also predicted to be pathological by NMD.

In F5, we also identified an *FRMD7* variant c.875 T > C:p.(Leu292Pro). Similarly, in F8, we identified a homozygous *TYR* variant c.1205G > A:p.(Arg402Gln) (Table 1) (see discussion for postulated implications).

### Clinical characteristics

**Figure 3:**
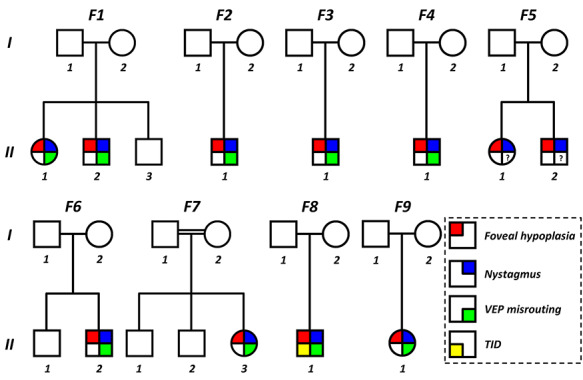
Pedigrees of families with SLC38A8 mutations. VEP = visual evoked potentials; TID = trans-illumination defects of the iris; ? = unknown (not performed)..

The pedigrees and summary of clinical characteristics of the families with *SLC38A8* mutations are shown in Fig. 3 and Table 2, respectively.

**Table 2:** Clinical characteristics of patients with *SLC38A8* mutations

Pedigree ID	Patient ID	Initial clinical diagnosis	Genetic diagnosis	Sex	Age (years)	Ethnicity	Refraction		BCVA^a^		Anterior Segment	Strabismus	Nystagmus^b^	Ultra-wide field imaging	OCT^c^	VEP
							RE	LE	RE	LE						
F1	F1:II-1	PAX6-related phenotype	*SLC38A8*	F	18	Korean	+2.00–3.00 Ax180	+2.00–3.00 Ax180	0.52	0.70	Normal	No	PFS	N/A	Grade 3	Chiasmal misrouting
F1	F1:II-2	PAX6-related phenotype	*SLC38A8*	M	15.8	Korean	−0.25 -3.00 Ax180	−0.50 -4.00 Ax180	0.70	0.52	Normal	Exotropia	DJ	N/A	Grade 3	Chiasmal misrouting
F2	F2:II-1	PAX6-related phenotype	*SLC38A8*	M	10	Korean	+2.75–3.50 Ax180	+3.25–2.75 Ax15	0.30	0.52	Normal	No	PPFS	N/A	Grade 3	Chiasmal misrouting
F3	F3:II-1	Ocular albinism	*SLC38A8*	M	0.6	Korean	+2.00–3.00 Ax180	+2.00–4.00 Ax180	CSM	CSM	Normal	No	BDJ	N/A	N/A	Chiasmal misrouting
F4	F4:II-1	Idiopathic infantile nystagmus	*SLC38A8*	M	6	Korean	+3.25–2.00 Ax180	+2.75–2.50 Ax180	0.40	0.40	Normal	No	BDJ	CMR	Grade 3	Chiasmal misrouting
F5	F5:II-1	PAX6-related phenotype	*SLC38A8*	F	14	Korean	+4.50–4.00 Ax170	+5.00–3.00 Ax180	0.30	0.52	Normal	No	BDJ	N/A	Grade 3	NA
F5	F5:II-2	PAX6-related phenotype	*SLC38A8*	M	12	Korean	+1.75–3.50 Ax180	+2.00–3.50 Ax180	0.30	0.52	Normal	Esotropia	BDJ	N/A	Grade 3	NA
F6	F6:II-2	PAX6-related phenotype	*SLC38A8*	M	27	Korean	+2.00–2.00 Ax180	+3.25–2.50 Ax10	0.52	0.40	Normal	Exotropia	PFS	CMR	Grade 3	Chiasmal misrouting
F7	F7:II-3	?FHONDA	*SLC38A8*	F	13	Turkish	+1.00–2.00 Ax180	+1.00–2.00 Ax180	0.78	0.64	Normal	Esotropia	PFS	N/A	Grade 3	Chiasmal misrouting
F8	F8:II-1	Ocular albinism	*SLC38A8*	M	28	Caucasian	+3.75–4.50 Ax180	+4.00–4.00 Ax180	0.60	0.60	TID	Esotropia	PAN	N/A	Grade 4	Chiasmal misrouting
F9	F9:II-1	Albinism	*SLC38A8*	F	36	Caucasian	+1.25–1.25 Ax160	+1.00–1.00 Ax180	0.64	0.60	TID	Exotropia	BDJ	N/A	Grade 4	Chiasmal misrouting

^a^BCVA reported in logMAR.

^b^All nystagmus was horizontal and conjugate with accelerating slow phases.

^c^Structural grading was based on the scheme previously described: Thomas M.G., Kumar A., Mohammad S. et al. Structural grading of foveal hypoplasia using spectral-domain optical coherence tomography a predictor of visual acuity? Ophthalmology 2011, 1653–1660.

Abbreviations: FHONDA = foveal hypoplasia with optic nerve decussation defects and anterior segment dysgenesis without albinism; F = female; M = male; RE = right eye; LE = left eye; BCVA = best corrected visual acuity; CSM = central steady and maintained; OCT = optical coherence tomography; VEP = visual evoked potentials; TID = trans-illumination defects of iris; BDJ = Bidirectional jerk; DJ = dual jerk; PPFS = pseudopendular with foveating saccades; PFS = pendular with foveating saccades; PAN = periodic alternating nystagmus; N/A = not available; CMR = concentric macular ring sign present.

Prior to genetic and VEP testing, the provisional clinical diagnoses are shown in Table 2. Only one family (F7) had a clinical diagnosis of possible FHONDA. Most families had a suspected diagnosis of *PAX6*-related phenotype (6/11 patients), ocular albinism (3/11 patients) or idiopathic IN (1/11 patients). F3:II-1 had fundus hypopigmentation and F8:II-1 had TID of the iris. All other patients did not have any evidence of cutaneous hypopigmentation, iris TID or fundus hypopigmentation. The best corrected visual acuity (VA) ranged between 0.30 and 0.78 logMAR with a median VA of 0.52 logMAR. The median spherical equivalent was +0.625 diopters (D) (range: −1.75 to 2.5D) in the right eye and +0.50D (range: −2.5 to 3.5D) in the left eye. All patients, except one (F9:II-1) had moderate-to-high with the rule astigmatism (WTR) (Table 2). Six of the patients (54%) had strabismus (Table 2). There was no evidence of ASD in any of the affected family members. All patients had nystagmus, FH and VEP crossed asymmetry similar to albinism suggestive of chiasmal misrouting (VEP was not available for family F5).

### Nystagmus characteristics

**Figure 4:**
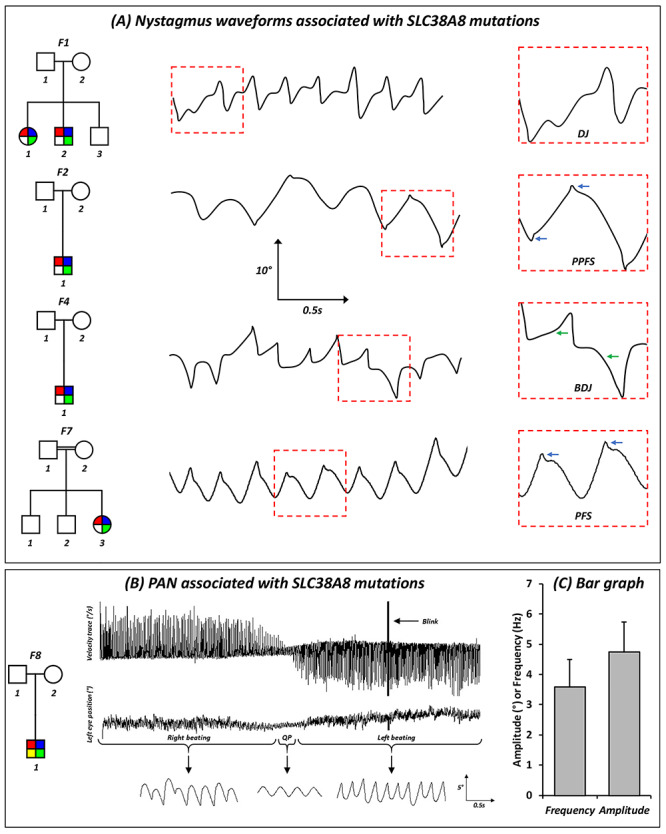
(**A**) Original eye movement recordings showing nystagmus characteristics and waveforms associated with SLC38A8 mutations. Blue arrows show foveating saccades and green arrow show accelerating slow phase, a characteristic of infantile nystagmus. (**B**) PAN associated with a SLC38A8 mutation in F8:II-1. Top panel shows the horizontal velocity trace (in degrees per second). The middle and bottom panels show the horizontal eye position of the left eye (degrees) in a compressed view and magnified views, respectively. The magnified traces show the different parts of the PAN cycle, initially right beating nystagmus followed by a short quiet phase, which has pendular nystagmus and subsequently switching to left beating nystagmus. The switch in direction of the PAN cycle is also readily seen in the velocity trace. For all eye movement traces—a deflection of the trace upwards signifies movement of the eyes to the right and a deflection downwards represents movement of the eye to the left. Scales for the magnified view are shown in the bottom panel. (**C**) Bar graph illustrating the mean frequency (Hz) and amplitude (°) in patients with SLC38A8 mutations. The error bars indicate the standard deviation (SD). Abbreviations: DJ = dual jerk; PPFS = pseudopendular with foveating saccades; BDJ = bidirectional jerk; PFS = pendular with foveating saccades.

All affected patients had horizontal conjugate nystagmus with infantile onset (within first 6 months after birth). Eye movement recordings revealed the characteristic accelerating slow phase seen in IN (Fig. 4). Bidirectional jerk nystagmus was the most common type of nystagmus seen (Fig. 4 and Table 2). Other waveforms included: dual jerk nystagmus (F1:II-2), pseudopendular with foveating saccades (F2:II-1) and pendular with foveating saccades (F1:II-1, F6:II-2 and F7:II-3). Periodic alternating nystagmus (PAN) was identified in F8:II-1 (Fig. 4). The PAN cycle had a periodicity of 190 s. Nystagmus frequency ranged between 2.0 and 4.7 Hz with a mean of 3.6 Hz. Nystagmus amplitude ranged between 3.7 and 6.7° with a mean of 4.7°. Plots of nystagmus amplitude and frequency are shown in Figure 4.

### Foveal hypoplasia

**Figure 5:**
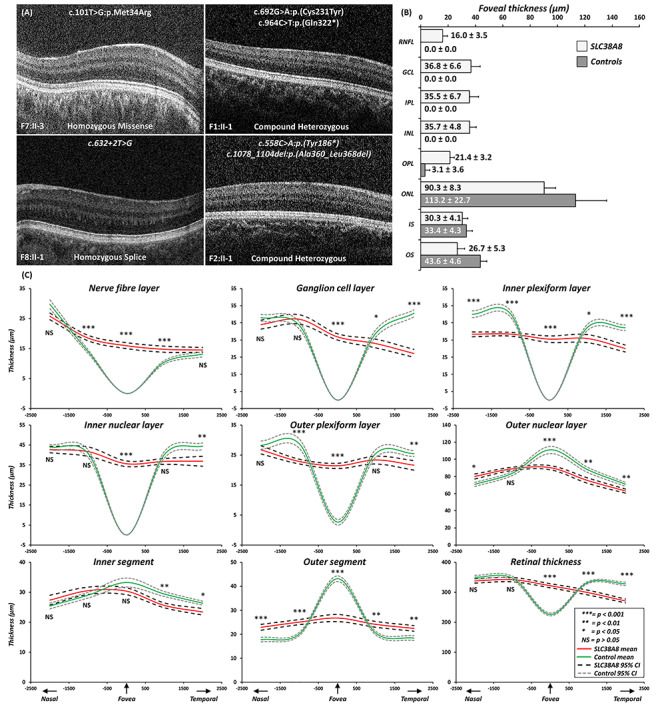
(**A**) Foveal tomograms associated with different SLC38A8 mutations. There is no evidence of a foveal pit or photoreceptor OS lengthening. In F1:II-1, F2:II-1 and F7:II-3, there some evidence of ONL widening, consistent with grade 3 foveal hypoplasia. In F8:II-1, there is no ONL widening which is characteristic of grade 4 foveal hypoplasia. (**B**) Bar graph showing the mean thickness for intra-retinal layers at the fovea for SLC38A8 patients and controls. The error bars indicate SD. (**C**) Mean thickness plots of each retinal layer with 95% confidence intervals for SLC38A8 patients (red) and controls (green). *X*-axis represents distance away from fovea in the nasal and temporal directions (in microns). For statistical comparisons thickness measurements were compared at the fovea, 1 and 2 mm from fovea in both the nasal and temporal directions. Levels of significance indicated alongside the relevant position (*** = *P* < 0.001; ** = *P* < 0.01; * = *P* < 0.05; NS = not significant (*P* > 0.05)). Abbreviations: RNFL = retinal nerve fibre layer; GCL = ganglion cell layer; IPL = inner plexiform layer; INL = inner nuclear layer; OPL = outer plexiform layer; ONL = outer nuclear layer; IS = inner segment; OS = outer segment.

On morphological assessment, none of the patients with *SLC38A8* mutations had a foveal pit or photoreceptor OS lengthening. The only OCT feature of foveal specialization observed was ONL widening (grade 3 FH). Thus, all affected patients had high grades of FH (either grade 3 or 4) (Fig. 5). The foveal retina was significantly thicker in the SLC38A8 group (mean ± SD: 322 ± 23.3 μm) compared with the control group (mean ± SD: 223 ± 14.8 μm; *P* = 3.42 × 10^−13^) (Fig. 5). The retinal layers RNFL, GCL, IPL and INL and OPL were significantly thicker in patients with *SLC38A8* mutations compared with controls (*P* < 0.0001, Fig. 5). The ONL and OS were significantly thinner in patients with *SLC38A8* mutations compared with controls (*P* < 0.0001, Fig. 5). There was no significant difference in IS thickness (*P* = 0.11).

In the parafoveal region, we find a nasotemporal asymmetry in thickness measurements between patients with *SLC38A8* mutations and controls. No significant difference was seen in total RT in the nasal parafovea (at both 1 and 2 mm away from the fovea), however, in the temporal parafovea (at both 1 and 2 mm), the SLC38A8 group had significantly reduced RT compared with controls (*P* < 0.0001). This was attributed to a significantly thinner GCL, IPL, INL, OPL, ONL and IS compared with controls (Fig. 5). Although differences in RNFL were noted at 1 mm both nasally and temporally, no significant (*P* > 0.05) differences were noted at 2 mm away from the fovea.

**Figure 6:**
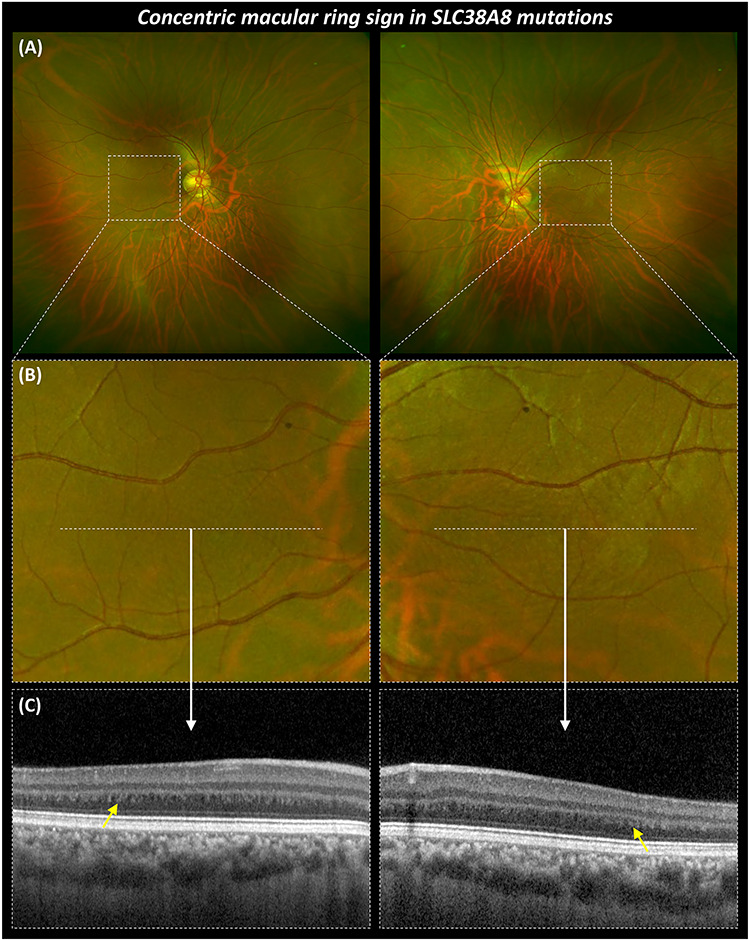
Multimodal imaging highlighting the CMR sign in SLC38A8 mutations. (**A**) Ultra-widefield fundus imaging from the right and left eyes of F6:II-2. (**B**) Magnified (5x) images show concentric rings surrounding expected location of fovea. (**C**) OCT scans showing foveal hypoplasia and highlighting (yellow arrow) the alternating hypo- and hyper-reflective vertical bands in the parafoveal Henle fibre layer.

Ultra-widefield imaging revealed the concentric macular ring (CMR) sign (Fig. 6) in four patients that had the imaging. Corresponding OCT scan showed the alternating hypo- and hyper-reflective vertical bands in the parafoveal Henle fibre layer (Fig. 6).

## Discussion

In this study, we describe the detailed genetic and phenotypical characteristics in patients with *SLC38A8* mutations. This is the first study describing the nystagmus characteristics and comprehensive retinal abnormalities in patients with *SLC38A8* mutations. Due to the rarity of this disorder, previous studies have been limited to case reports (5) or series (4,6,7) with partial phenotypic data. Thus, it has not been possible to systematically investigate the phenotypical characteristics associated with *SLC38A8* mutations. Over the last 7 years since the identification of *SLC38A8* mutations causing FHONDA (3), a total of 17 *SLC38A8* mutations have been described. We describe a further 16 *SLC38A8* mutations and thus expand the genotypic spectrum including splice mutations associated with this disorder. Moreover, we describe for the first-time patients from Korean descent with *SLC38A8* mutations. Taking together the mutations, we describe in this study with those reported in the literature, we find that the sixth transmembrane domain in SLC38A8 is most frequently affected from missense mutations. We show that this is a highly conserved region of the SLC38A8 protein (Supplementary Material, Fig. S1), suggesting that the residues within this domain are critical to the normal functioning of the protein. Using three-dimensional (3D) modelling, we also predict that this domain is an alpha-helix residing within the core of this putative sodium-coupled neutral amino acid transporter.

**Figure 7:**
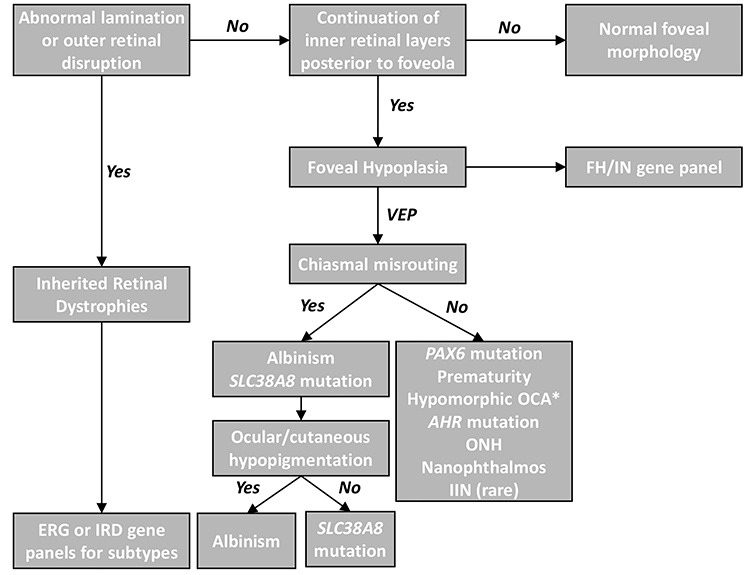
Clinical diagnostic algorithm for abnormal retinal development. The hallmark of typical foveal hypoplasia is continuation of inner retinal layers posterior to the foveola. Outer retinal changes or abnormal lamination suggests an inherited retinal dystrophy (IRD). Electroretinogram (ERG) or IRD gene panels can differentiate between the IRD subtypes. FH or IN gene panels can readily differentiate the genetic subtypes that can cause FH. In the absence of genetic panels, VEP is helpful in broadly differentiating the diagnoses based on chiasmal misrouting. In some cases of albinism* (hypomorphic mutations or carriers), normal VEP responses maybe present. ONH = optic nerve hypoplasia, IIN = idiopathic infantile nystagmus.

Diagnosing FHONDA is challenging. This is partly due to its rarity and secondly our knowledge of the phenotypic spectrum is limited. In the original descriptions of FHONDA, ASD features as a prominent part of the phenotype (2). However, subsequent reports (4,5,6,7) and certainly in our cohort of patients, we did not identify any cases of ASD. It was previously hypothesized that ASD is only seen in severe cases of FHONDA associated with deletions (5). However, in our cohort, we had five families (F2, F3, F6, F8 and F9) with predicted truncating mutations with no ASD. Thus, ASD likely represents a minor association with this disorder exhibiting variable expressivity. Among Jewish ethnicities a founder mutation (c.95 T > G; p.(Ile32Ser)) has been described (4,7), of the nine affected individuals (from five families described by Weiner et al. (7) and one family described by Perez et al. (4)) only one (11%) had mild posterior embryotoxon (7). Considering that the normal prevalence of posterior embryotoxon is 6.8% in the general population and 22.5% in the younger age groups (8), this could also be a phenocopy. In one of our patients (F8:II-1) (1/11), we identified mild iris TID. However, careful examination of all variants in our nystagmus panel revealed a homozygous *TYR* variant (c.1205G > A; p.(Arg402Gln)), which could explain the TID. Subtle TID can also been seen in carriers of albinism. Similarly, in F5:II-1 and F5:II-2, we also identified an *FRMD7* variant (c.875 T > C:p.(Leu292Pro)) (see Table 1), which has previously been reported (9). Thus both these mutations could also be contributory to the phenotype and may represent dual diagnoses. However, there have been no cases of grade 3 or 4 FH reported with *FRMD7* mutations (10). Consistently in all affected patients with *SLC38A8* mutations, we observe: (1) high grade FH (grade 3 or 4), (2) IN and (3) chiasmal misrouting detected on VEP. The median VA of our patients with SLC38A8 mutations was 0.52 logMAR. This is similar to the median VA seen in albinism (0.50 logMAR) (1). In *PAX6* mutations, the median VA ranges between 0.48 and 1.52 logMAR depending on the type of mutation (33). In addition to FH, *PAX6* mutations can be associated with keratopathy, cataracts, glaucoma and optic nerve hypoplasia thus resulting in much more reduced vision compared with *SLC38A8* mutations and albinism (11). In our cohort of patients with FH and IN, *SLC38A8* mutations only accounted for 1% (3 families/300 unrelated patients) and 2.8% (6 families/211 unrelated patients) in a UK and a Korean tertiary paediatric ophthalmology department respectively. In a large French cohort of suspected cases of albinism, *SLC38A8* mutations were identified in 0.4% (4/990) cases (6). This raises the possibility that *SLC38A8* mutations may have a higher disease burden in Korea. This could be related to the high allele frequency of c.995dupG variant in Korean and Japanese populations (0.0026 in gnomAD Korean population and 0.0015 in 4.7 K ToMMo Japanese). Based on our initial clinical examination, most common provisional diagnoses were *PAX6*-related phenotype and ocular albinism. This was due to the significant phenotypic overlap between these disorders. Typically, *PAX6* mutations are associated with aniridia, however, recent studies have reported cases with milder phenotypes with minimal to no iris abnormalities (12,13). Thus from the initial clinical examination (prior to VEP testing), in the absence of ocular or cutaneous hypopigmentation, *PAX6*-related phenotype could be considered a differential diagnosis. However, once chiasmal misrouting is established on VEP testing, only albinism and *SLC38A8* mutations are considered as differentials. Though hypopigmentation (ocular and/or cutaneous) is a characteristic of albinism, significant phenotypic heterogeneity is associated with albinism (1), thus making the diagnosis more challenging. We have devised a diagnostic algorithm, which can be helpful in prioritizing differential diagnoses and investigations (Fig. 7). The advent of targeted re-sequencing IN panels (14,15) can potentially obviate the need for the battery of tests these patients often undergo (14). However, the main disadvantages of next generation sequencing (NGS) are the lag time to obtaining results and, in some instances, determining pathogenicity of variants. Nevertheless, NGS can be a valuable frontline diagnostic tool as demonstrated in this study where we revised our initial diagnosis in 10/11 cases, it also provides deeper insight into the phenotypes seen (as demonstrated for example with patient F8:II-1) and opportunities for genetic counselling.

We describe for the first time the nystagmus characteristics seen in patients with *SLC38A8* mutations. Similar to other forms of IN (16), the nystagmus is horizontal conjugate with the characteristic increasing slow phase velocity. Kumar et al. showed that albinism had a higher proportion of jerk waveforms in comparison with *FRMD7*-related IN (17). In most patients with *SLC38A8* mutations, we observe a jerk nystagmus. However, we also describe pendular nystagmus with foveating saccades in three patients (F1:II-1, F6:II-2 and F7:II-3). The periodicity and features of PAN we describe in F8:II-1 are similar to what has been documented with albinism and *FRMD7* mutations (17,18,19). Previous work has shown that the frequency of nystagmus is significantly different between albinism (mean = 3.3 Hz) and *FRMD7* mutations (mean = 4.3 Hz), in our study, we find that the mean frequency in the SLC38A8 group is 3.6 Hz. Thus, within the same range as other forms of IN. The shared characteristics between different forms of IN has been hypothesized to arise from failure of the smooth-pursuit damping circuitry to properly calibrate during early development (20,21). Recent work by Winkelman et al. show that congenital stationary night blindness (CSNB) in humans and a nyx mouse model (*nob* mice) have horizontal nystagmus with a frequency of 4–7 Hz, interestingly the ON direction-selective ganglion cells (GC) that project to the accessory optic system had electrical oscillations at the same frequency as the ocular oscillations. They propose the origin for these oscillations is presynaptic to GC and arise from AII amacrine cells (22). These cells generate intrinsic oscillations (23) that drive oscillatory firing of ON-and OFF-GCs in antiphase (24). Moreover, pharmacological modulation of the oscillatory frequency of these cells also results in modulation of the nystagmus frequency in *nob* mice (22). SLC38A8 is expressed within the plexiform layers in the retina (3), however, it is not known if it localizes to a specific retinal cell type. Further work is needed to understand whether the findings by Winkelman et al. (22) can be extended to other forms of IN including nystagmus associated with *SLC38A8* mutations.

Most of our patients (10/11) had significant WTR astigmatism. This is an interesting observation we report and comparison with previous cases (Supplementary Material, Table S1), we find that all have significant WTR astigmatism. Taken together, this suggests that patients with *SLC38A8* mutations almost always have significant WTR astigmatism. Previous work on the distribution of refractive errors between idiopathic IN and albinism showed that albinism was more commonly associated with significant astigmatism (primarily WTR) when compared with idiopathic nystagmus (25). Furthermore, longitudinal data from IN syndrome (INS) show meridional emmetropization in children with INS and WTR astigmatism (26).

Using OCT, we have been able to quantify the degree of arrested retinal development in patients with *SLC38A8* mutations for the first time. Interestingly, all the patients in our study had high grades of FH (either grade 3 or 4) (27). Reviewing literature, we are able to identify OCT scans available (*n* = 9 foveal scans) in a few of the *SLC38A8* mutation reports (3,4,5,7). We graded the available scans and find that all patients had either grade 3 (*n* = 6) or grade 4 FH (*n* = 3) (3,4,5,7). Taken together this means that the retinal phenotype is more severe in *SLC38A8* mutations suggestive of an earlier foveal developmental arrest, hence, we see no signs foveal pit formation or cone outer segment lengthening. In albinism, *PAX6* mutations and other isolated cases we find a larger spectrum of FH (grades 1–4) (27). We also find that the foveal outer segment thickness reduced significantly in the SLC38A8 group, which morphologically is seen as a lack of OS lengthening and thus cone specialization. Previous work in albinism has shown that the OS length is a surrogate marker of peak foveal cone density (28). Thus, we hypothesize that the reduced OS length in *SLC38A8* mutations likely represents a reduced foveal cone density. This is consistent with SLC38A8 immunohistochemistry data, which show protein expression in plexiform and photoreceptor layers (3). In the parafovea, we report nasotemporal asymmetry of total retinal thickness with the temporal parafovea significantly thinner in the SLC38A8 group compared with controls. This is due to the asymmetrical developmental sequence within the retina, the temporal (temporal to incipient fovea) and peripheral retina are slower to develop and differentiate compared with the central retina (29). As the fovea pit forms, there is centrifugal displacement of the inner retinal layers (27,30), however, this developmental event does not occur in *SLC38A8* mutations. Further comparisons with other forms of FH are important to differentiate whether different mechanisms for arrested retinal development exist.

We also report for the first time that the CMR sign is present in *SLC38A8* mutations. This sign was initially identified using infrared reflectance imaging and reported in FH associated with albinism and aniridia (31). Recently, it was also shown using ultra-wide field imaging and provided evidence to suggest that the concentric rings seen are due to alternating hypo- and hyper-reflective vertical bands in the parafoveal Henle fibre layer. Moreover, the horizontal diameter of the largest outer ring significantly correlates to the FH grading and VA (32). The presence of CMR in another disorder (*SLC38A8* mutations) associated with FH expands the spectrum of diseases associated with this sign.

In conclusion, we expand on the genotypic and phenotypic spectrum of *SLC38A8* mutations. We describe for the first time the nystagmus characteristics and quantify the retinal abnormalities associated with this disorder. *SLC38A8* mutations share similar nystagmus characteristics to other forms of IN suggesting a common pathophysiological mechanism. However, the degree of arrested retinal development is more severe and quantitative measurements show reduced thickness of the cone photoreceptor outer segments indicative of reduced foveal cone photoreceptor density.

## Methods

### Genotyping

As part of a large-scale genotyping project, patients with IN (*n* = 511) were recruited at one centre in the UK (University Hospitals of Leicester) and two centres in Korea (Yonsei University College of Medicine and University of Ulsan College of Medicine) over a period of 5 years (2014–2019). Informed consent was obtained from all participants involved. The study was approved with a local ethics committee and all aspects of the study were conducted in accordance with the Tenets of the Declaration of Helsinki.

The details of our pilot genotyping project have been previously described (14,15). Briefly, this included extraction of genomic DNA from saliva samples (DNA Genotek OG-500 and OG-575 Oragene saliva kits (Ottawa, Ontario, Canada)) or peripheral blood and subsequently performing targeted NGS using our nystagmus gene capture panels or whole exome sequencing. Our nystagmus gene panels included all known nystagmus genes described in the literature (14,15). Our initial panels were revised to also include the recently identified nystagmus gene, *AHR*, mutations of which we have described to be causative of IN and FH (33). Our aim was to identify the prevalence of *SLC38A8* mutations in our cohorts and describe the phenotypical characteristics.

### Variant filtering and classification

The pathogenicity of missense variants was predicted using *in silico* prediction algorithms, including SIFT, PolyPhen2, FATHMM and CADD (34). Splice site analysis was performed using the MaxEntScan, NNSPLICE (Neural Network Splice Prediction), Human Splice Finder, GeneSplicer and SpliceFinder-like algorithms implemented in the Alamut Visual software (Interactive Biosoftware, Rouen, France). Segregation analysis was performed in all families except F5 (father (F5:I-1) was not available for genetic testing). The interpretation of variants followed the five-tier classification system recommended by the ACMG and Genomics and the Association for Molecular Pathology (Table 1) (35). The exact 3D structure of SLC38A8 protein is unknown, thus we used I-TASSER v5.1 (36,37) deployed within our high-performance computing cluster, ALICE to predict the SLC38A8 protein 3D structure and map the amino acid substitutions from missense mutations. I-TASSER is considered one of the best protein structure prediction algorithms, which uses meta-threading and *ab initio* modelling methods to match fragments of the protein sequence onto the 3D structure of other solved proteins deposited in the protein data bank (PDB: https://www.rcsb.org/) (36,37). Subsequently, we also modelled the missense mutations using STRUM (15) predicting stability changes (ΔΔ*G*) of *SLC38A8* missense mutations. Where the Gibbs free energy gap difference (ΔΔ*G* = Δ*G_m_* – Δ*G_w_*) between the mutant (Δ*G_m_*) and wild type (ΔG_w_) protein is a measure of how the mutation affects protein stability. A ΔΔ*G* below zero means that the mutation causes destabilization; otherwise, it induces stabilization (38).

### Phenotyping

All subjects underwent detailed ophthalmic examination and investigations to determine the phenotypical characteristics. This included a full orthoptic assessment, anterior segment slit lamp examination to identify TID of the iris and ASD. Fundoscopy and optical coherence tomography (OCT) was performed to identify FH. OCT was performed using either Envisu C2300 (Leica Microsystems, Wetzlar, Germany) or Heidelberg Spectralis (Heidelberg Engineering, Heidelberg, Germany). A 3D horizontal raster scan protocol (AxB scans: 600 x 80; scan window 12 x 8 mm), centred at the macula (39,40). The central foveal B-scan was selected based on the scan with the deepest foveal pit. In the absence of a foveal, pit features of cone photoreceptor specialization were used (27,41). Conversion factors for both lateral (0.989) and axial (0.975) measurements were applied to Spectralis tomograms to allow translation of quantitative data across our OCT platforms (42). The retinal layers were segmented using a semi-automated ImageJ macro as described (39,40). Retinal thickness measurements obtained were: nerve fibre layer (NFL), GC layer (GCL), inner plexiform layer (IPL), inner nuclear layer (INL), outer plexiform layer (OPL), outer nuclear layer (ONL), inner segment (IS), outer segment (OS) and total retinal thickness (RT). For purposes of statistical comparisons, measurements were extracted from five positions: foveal centre, 1 and 2 mm away from the fovea in the nasal and temporal directions. We have previously shown good reliability and reproducibility of retinal thickness measurements from patients with nystagmus (43). Control data (age and gender matched) (*n* = 18; 12 male, 6 female, mean age ± SD 14.9 ± 7.1) were obtained from the Leicester OCT normative dataset. The control group had no known eye pathology, systemic disease or previous intraocular surgery. There was no history of prematurity in both the SLC38A8 and control group. OCT images were graded using the Foveal Hypoplasia Grading Scale (27) with Grade 1 subdivided into 1a and 1b as per Wilk et al. (44). This has prognostic value in preverbal children with nystagmus (45). VEP (ISCEV standards) were used to assess misrouting of retinal GC axons at the optic chiasm. All patients except F5:II-1 and F5:II-2 (Table 2) underwent VEP recordings as per ISCEV standards (46). Patients had a multichannel VEP with either a pattern onset/offset stimulus (F7:II-3, F8:II-1 and F9:II-1) or a flash stimulus (F1-F4 and F6, see Table 2). Eye movement recordings were obtained using an infra-red video pupil tracker (Eyelink II, SR Research, Osgoode, Canada and SLMED, Seoul, Korea). Nystagmus waveform at primary position was classified into 12 categories (Dell’Osso and Daroff Classification) (47). An extended fixation task was utilized for detection of PAN as described (19). To assess whether the CMR sign (31,32,48) is present in patients with *SLC38A8* mutations and FH, we obtained ultra-widefield fundus images (Optos PLC, Dunfermline, UK) in four eyes of two patients with *SLC38A8* mutations.

### Statistical analyses

Statistical analysis was performed using IBM SPSS Statistics software (version 24, IBM Corp.). A generalized linear mixed model was used to determine significant differences in retinal layer thickness measurements between patients and controls. Within the model, eye (left vs right) was assigned as a repeated measure, fixed factor was the diagnosis (patients vs controls) and random factors included age, gender, ethnicity and refraction. Bonferroni correction was applied for multiple testing. *P* < 0.05 was considered statistically significant.

## Supplementary Material

Supplementary_Figure_2_splice_analysis_ddaa166

Supplementary_table_1_ddaa166
