# Supplementary material for: SLC38A8 mutations result in arrested retinal development with loss of cone photoreceptor specialization
Source: Hum Mol Genet. 2020 Aug 3;29(18):2989–3002. doi: 10.1093/hmg/ddaa166 (PMC7645707; doi:10.1093/hmg/ddaa166)

Supplementary Figure 2: c. 1214+5G>C – splice analysis

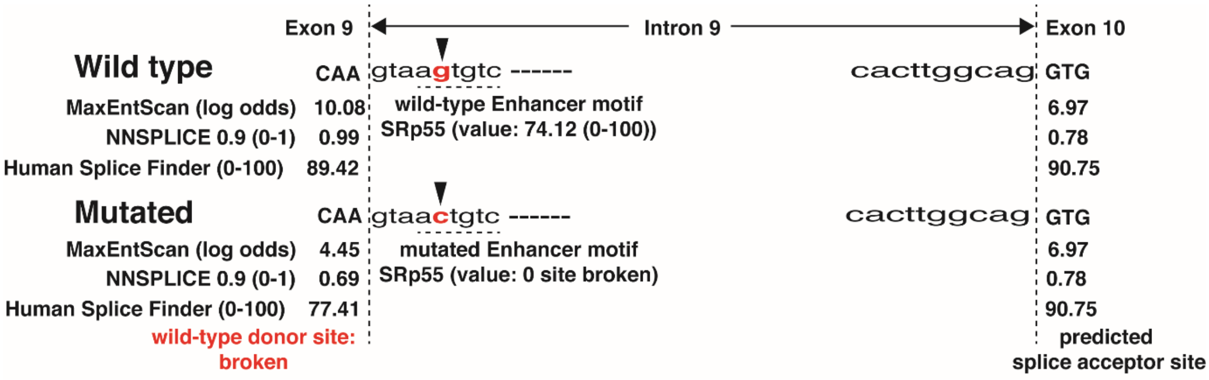

Supplement: Supplementary_Figure_2_splice_analysis_ddaa166 [file supplementary_figure_2_splice_analysis_ddaa166.pdf]
