# Supplementary material for: SLC38A8 mutations result in arrested retinal development with loss of cone photoreceptor specialization
Source: Hum Mol Genet. 2020 Aug 3;29(18):2989–3002. doi: 10.1093/hmg/ddaa166 (PMC7645707; doi:10.1093/hmg/ddaa166)
Supplement: Supplementary_table_1_ddaa166 [file supplementary_table_1_ddaa166.docx]

**Supplementary table 1: Refractive errors in patients with SLC38A8 mutations previously reported in the literature**

|  | Mutations | Refractive errors | References |
| --- | --- | --- | --- |
| 2 families | c.1002delG p.(Ser336Alafs*15) homo  c.1234G>A p.(Gly412Arg) homo  (these mutations were reported in Poulter et al. 2013) | -2.00~-5.50 astigmatism (WTR) | van Genderen et al. Br J Ophthalmol 2006 |
| 2 families | 16q23.3-24.1 (mutations reported in Poulter et al. 2013) | -2.00 ~-5.50 astigmatism (WTR) | Al-Araimi et al. Mol Vis 2013 |
| 7 families | c.707T>A:p.(Val236Asp) homo  c.1002delG:p.(Ser336Alafs*15) homo  large deletion / c.1234G>A:p.(Gly412Arg) hetero  c.1029delG:p.(Leu344Cysfs*7) homo  c.101T>G:p.(Met34Arg) homo  c.697G>A:p.(Glu233Lys) homo  c.598C>T:p.(Gln200*) / c.845_847delCTG:p.(Ala282del) | NA | Poulter et al. Am J Hum Genet 2013 |
| 3 family | c.95T>G:p.(Ile32Ser) homo | All patients had astigmatism. | Perez et al. Eur J Hum Genet 2014 |
| 1 family | c.848A>G:p.(Asp283Ala) homo | +5.00 -2.00 Ax180 (WTR) | Toral et al. Mol Genet Genomic Med 2017 |
| 4 families  1 family | c.95T>G:p.(Ile32Ser) homo  c.95T>G:p.(Ile32Ser) / c.490_491del: p.(Leu164Valfs*41) hetero | NA  NA | Weiner et al. Exp Eye Res 2020 |

NA = not available

WTR = with the rule astigmatism
